# Phylogeographic Structure of a Tethyan Relict *Capparis spinosa* (Capparaceae) Traces Pleistocene Geologic and Climatic Changes in the Western Himalayas, Tianshan Mountains, and Adjacent Desert Regions

**DOI:** 10.1155/2016/5792708

**Published:** 2016-05-24

**Authors:** Qian Wang, Ming-Li Zhang, Lin-Ke Yin

**Affiliations:** ^1^Key Laboratory of Biogeography and Bioresource in Arid Land, Xinjiang Institute of Ecology and Geography, Chinese Academy of Sciences, Urumqi, Xinjiang 830011, China; ^2^University of Chinese Academy of Sciences, Beijing 100049, China; ^3^Institute of Botany, Chinese Academy of Sciences, Beijing 100093, China

## Abstract

Complex geological movements more or less affected or changed floristic structures, while the alternation of glacials and interglacials is presumed to have further shaped the present discontinuous genetic pattern of temperate plants. Here we consider* Capparis spinosa*, a xeromorphic Tethyan relict, to discuss its divergence pattern and explore how it responded in a stepwise fashion to Pleistocene geologic and climatic changes. 267 individuals from 31 populations were sampled and 24 haplotypes were identified, based on three cpDNA fragments (*trn*L-*trn*F,* rps*12-*rpl*20, and* ndh*F). SAMOVA clustered the 31 populations into 5 major clades. AMOVA suggests that gene flow between them might be restricted by vicariance. Molecular clock dating indicates that intraspecific divergence began in early Pleistocene, consistent with a time of intense uplift of the Himalaya and Tianshan Mountains, and intensified in mid-Pleistocene. Species distribution modeling suggests range reduction in the high mountains during the Last Glacial Maximum (LGM) as a result of cold climates when glacier advanced, while gorges at midelevations in Tianshan appear to have served as refugia. Populations of low-altitude desert regions, on the other hand, probably experienced only marginal impacts from glaciation, according to the high levels of genetic diversity.

## 1. Introduction

An essential purpose of biogeography is to comprehend the mechanisms resulting in the current distribution patterns of organisms during their evolutionary histories [1, 2]. Events since the late Paleogene, especially the Quaternary geologic and climatic changes, likely had a far-reaching influence on the spatial geographic structure and genetic diversification of temperate species [3, 4]. After the Oligocene, the inland Tethys or Paratethys Sea retreated, accompanied by an increasingly droughty climate, resulting in elaboration of the xerophytic Tethyan Flora [5, 6]. From the Neogene to Quaternary, with the increasing aridification and uplift of the Himalayas, Tibet Plateau, and Tianshan Mountains [7, 8], the originally continuous arid Tethyan Flora gradually evolved into the North Temperate, Mediterranean, West Asian-Central Asian, Central Asian, and Himalayan elements of today [9, 10]. Complex geological movements more or less affected or changed floristic structures, while rapid climatic shifts likewise impacted plant life worldwide during this time. Significantly, the alternation of glacials and interglacials during the Pleistocene is presumed to have played a vital role in further shaping the present discontinuous genetic pattern for a majority of temperate plant species in the Northern Hemisphere [6, 11, 12].

Phylogeography, a discipline which began more than 20 years ago, has mostly considered the response of geographic distribution and genetic structure of current species to historical events and the cycles of glaciation [13–16]. Chloroplast DNA (cpDNA), suitable for reconstructing phylogenetic patterns of plant species, is effective in exploring and estimating phylogeographical history of temperate plants caused by climatic change [17–20]. Recent scholars have concentrated on the refuge during the Last Glacial Maximum (LGM) and range expansion during inter- or postglacial periods [21–24]. With the most focus on mountain plants in Europe, North America, and East Asia [25–27], phylogeographic patterns and demographical history of eremophytes in arid Northwestern China are also receiving increasing attention [28–30].

We use* Capparis spinosa*, a typical xerophyte, to verify the hypothesis of Pleistocene historical influence through phylogeographic analysis and discuss how its spatial genetic structure responded to paleogeologic and paleoclimatic changes. The perennial creeping subshrub is a type member of a relatively large genus of the Capparaceae, with a natural distribution in the Mediterranean, West, and Central Asia [31, 32]. In arid Western China, there is only one single genus (*Capparis* Tourn. ex L.) with one single species (*C. spinosa* L.) belonging exclusively to this family [33].* C. spinosa* ranges over the Western Himalayas, Eastern Pamir Plateau, Tianshan Mountains, Turpan-Hami Basin, and the Hexi Corridor. Previous studies on* Capparis* have paid more attention to autecology, phenology, quantitative morphology, and reproductive ecology [34–37]. Molecular studies related to the genetic variation of the species are little explored [38, 39] and have not considered genetic divergence from a phylogeographic point of view.

Here we employed three maternally inherited cpDNA spacers,* trn*L-*trn*F,* rps*12-*rpl*20, and* ndh*F, to trace historical divergence patterns of* C. spinosa*. We design to address the following concrete issues. (1) According to phylogenetic relationships yielded by cpDNA sequence variation, what is the genetic pattern of the species? (2) When and how have allopatric divergence and genetic differentiation of this xerophyte been influenced by glacial-interglacial cycles associated with geologic and climatic changes?

## 2. Materials and Methods

### 2.1. Population Sampling

A total of 267 individuals of* C. spinosa* from 31 natural populations were investigated in the present study (Table 1), throughout almost the entire geographic distribution of the species in Eastern Central Asia, including Xinjiang, western Gansu, and western Tibet. For each population, leaf samples were collected from 5 to 12 mature and large perennial individuals separated by at least 100 m, in order to avoid inbreeding and clones. The latitude, longitude, and altitude at each collection center were measured using a global positioning system (GPS) receiver. About 10 g of fresh leaf materials was gathered per individual, dried in silica gel in the field, and stored at −20°C in the laboratory.* Capparis bodinieri* Lévl. was chosen as an outgroup for the phylogenic study, based on previous taxonomic studies on* Capparis* [31]. Voucher specimens for all individuals were deposited in the Herbarium of the Xinjiang Institute of Ecology and Geography, Chinese Academy of Sciences (XJBI).

### 2.2. DNA Extraction, Amplification, and Sequencing

Silica-gel-dried leaf tissues were ground into powder in liquid nitrogen with a Fastprep-24 device (Sample Preparation System, MP Biomedicals, USA). Total genomic DNA was extracted from about 50 mg of powdered tissues using a modified 2x CTAB method [40]. Through preliminary screening from eighteen pairs of primers (see Table S1 in Supplementary Material available online at http://dx.doi.org/10.1155/2016/5792708), three cpDNA regions,* trn*L-*trn*F,* rps*12-*rpl*20, and* ndh*F (329F, 927R), were found because they have more sequence variation in individuals among populations. Polymerase chain reaction (PCR) amplification and DNA sequencing were performed using primer pairs for these three regions. PCRs were carried out in 30 *μ*L reaction volumes including 1.5 *μ*L of 10x buffer, 2 *μ*L of 25 mmol/L MgCl_2_, 2.2 *μ*L of 50 ng/*μ*L each primer (Sangon, Shanghai, China), 3 *μ*L of 2.5 mmol/L dNTP, 0.3 *μ*L of 5 U/*μ*L* Taq* DNA polymerase, and 1.0 *μ*L of 5 ng/*μ*L genomic DNA. PCR amplification was run on a Gene-Amp PCR system 9700 DNA Thermal Cycler (Applied Biosystems, Foster City, CA, USA) with parameters set as follows: an initial start at 94°C with 4 min; followed by 30 cycles of 94°C with 30 s, then 59°C, 52°C, and 52°C, respectively, for* trn*L-*trn*F,* rps*12-*rpl*20, and* ndh*F with 30 s and 72°C with 90 s; plus a subsequent additional extension at 72°C with 10 min. Sizes of PCR products were visualized on 1% TAE-agarose gel electrophoresis. All PCR products were purified with PCR product purification kit (Shanghai SBS, Biotech Ltd., China) and then sequenced in both directions for* trn*L-*trn*F,* rps*12-*rpl*20, and* ndh*F using an ABI Prism 3730 xl automated sequencer in Sangon Biological Engineering Technology & Service Co., Ltd., Shanghai, China.

### 2.3. Data Analysis

The chloroplast DNA sequences were edited by SeqMan (Lasergene, DNASTAR Inc., Madison, Wisconsin, USA). All sequences were aligned with Clustal X version 1.81 [41], then refined, and adjusted. All resulting cpDNA sequences identified by nucleotide variations, signifying different haplotypes, were deposited in GenBank. The corresponding accession numbers are KU940216–KU940218 for* trn*L-*trn*F, KU866560–KU866562 and KU940220 for* rps*12-*rpl*20, and KU866548–KU866558 and KU940221–KU940223 for* ndh*F (329F, 927R); and accessions numbers of* C. bodinieri* as the outgroup are KU940219 for* trn*L-*trn*F, KU866563 for* rps*12-*rpl*20, and KU866559 for* ndh*F (329F, 927R). Different cpDNA haplotypes were identified by DnaSP 5.0 [42]. Geographic distribution of detected haplotypes was mapped using ArcMap 9.3 (ESRI, Redlands, CA, USA). Phylogenetic relationships among cpDNA haplotypes were associated via a median-joining network (MJN) method [43], which was constructed using Network 4.6.1.0 (available at http://www.fluxus-engineering.com/sharenet_rn.htm).

To maximize the differences of haplotype composition between geographical locations, spatial analysis of molecular variance algorithm was conducted using SAMOVA 1.0 procedure. This method divided the sampled populations into inferred partition (*K* groups) based on genetic differentiation and geographical homogeneity [44]. We initially set the *K* values ranged from 2 to 12 by repeated simulated annealing process until largest *F*_CT_ index was obtained. However, when *K* ≥ 6, at least one of the groups contained only one population, implying that information on the group structure would have been disappearing [45–48]. To maintain the grouping pattern and exclude configurations with a single population in any of the groups, finally valid range of *K* was set as 2–5.

Parameters of Nei's diversity, containing haplotype diversity (*H*_*d*_) and nucleotide diversity (*π*), were computed in Arlequin 3.1 program [49, 50]. To evaluate genetic variation within populations, among populations within groups, and among groups, AMOVA (analysis of molecular variance) was performed in the program Arlequin version 3.1 [50], and significance tests were conducted using 1,000 permutations. The statistics of average gene diversity and population differentiation, that is, the average gene diversity within populations (*H*_*S*_), total gene diversity across all populations (*H*_*T*_), the number of substitution types (*N*_ST_), and genetic differentiation over populations (*G*_ST_), were calculated using Permut version 1.0 [51] (available at http://www.pierroton.inra.fr/genetics/labo/Software/PermutCpSSR/index.html). To test for the existence of phylogeographical structure, the comparison of *G*_ST_ versus *N*_ST_ was conducted using Permut based on 1,000 random permutations of haplotypes across populations. If *N*_ST_ is significantly higher than *G*_ST_, then genealogically closely related haplotypes tend to occur together within populations, indicating the presence of phylogeographical structure [51]. Significance of *P* values was examined by the *U* Test using the Haplonst procedure [51].

To estimate the divergence times of cpDNA haplotypes, the BEAST version 1.6.1 [52] was carried out for lineage analysis, following a Bayesian relaxed molecular clock. A Bayesian Markov Chain Monte Carlo (MCMC) procedure was run for 20,000,000 generations with a coalescent-based tree priority rule and a HKY substitution model. Given the uncertainty of the cpDNA mutation rate, the normal distribution priority with a mean value of 2 × 10^−9^ s s^−1^ yr^−1^ (substitutions per site per year) and a SD of 6.08 × 10^−9^ for most angiosperm species were adopted as a criterion for the analytical operation [24, 53], to cover the rate variation within the 95% range of the distribution for the present calculation of molecular divergence times of genotypes. The combined parameters were validated in Tracer version 1.5 [52] to check whether the parameter value was greater than 1 and the effective sample size value was larger than 200, as an indication that these sequence data were suitable for a relaxed molecular clock model. Trees were finally compiled in FigTree 1.3.1. The statistical support of clades depended on Bayesian posterior probability.

To investigate spatial genetic patterns of geographical distance among populations for each defined regional group, genetic landscape shape analysis was performed using Alleles In Space [54]. Primarily, a connective Delaunay triangulation network was constructed based on the geographic coordinates of all sampled points, according to the Watson (1992) and Brouns (2003) methods [55, 56]. Secondly, the average genetic distances resulting from the cpDNA sequence data were calculated among populations in the network. Afterwards, the genetic distance matrix was interpolated onto spatial grids of the connective network, forming a landscape shape. A final three-dimensional surface plot was generated, where the abscissa and ordinate signified geographical coordinates and the vertical coordinate were equivalent to genetic distance.

Possible demographic historical expansions for defined groups were inferred by mismatch distribution analysis using Arlequin with 1,000 parametric bootstrap replicates [57]. Populations that have experienced recent expansion are expected to have a unimodal curve in the pairwise mismatch distribution, whereas stable populations should have a bi- or multimodal shape [58, 59]. Neutrality tests (Tajima's *D* and Fu's *F*_*s*_ statistics) [60, 61] were used to detect further evidence for probable historical processes [62, 63]. A positive or negative *D* value (not 0) may imply that a population underwent expansion or bottlenecks [64], while a negative *F*_*s*_ value probably indicates a recent population expansion [61].

To explore the potential distribution changes of the species between the present-day and the Last Glacial Maximum (LGM; approximately 18–21 ka), the ecological niche modeling was performed in MAXENT version 3.3.1 [65], following the maximum entropy algorithm. We input an available coordinate set of occurrence points from the species' entire geographical distribution in Eastern Central Asia based on the Chinese Virtual Herbarium (http://www.cvh.org.cn/cms/) and our field survey locations (Table 1). The current climatic envelopes were predicted based on WorldClim dataset [66] and the LGM layers in accordance with the Model for Interdisciplinary Research on Climate (MIROC) [67] and the Community Climate System Model (CCSM) [68]. We acquired 19 BIOCLIM variables to model ecological niche at 2.5 arcmin resolution from the WorldClim database (available at http://www.worldclim.org/download/). To avoid the potential problems of overfitting [69], we removed the highly correlated bioclimatic variables, retaining nine least correlated ones. Model performance was identified by the area under the receiver operating characteristic curve (AUC). The default parameters to form ecological niche modeling were chosen as follows: regularization multiplier = 1.0, percentage of the data set for train the model = 75%, percentage for testing = 25%, and the threshold for available presence data = 10 percentiles. In addition, a Jackknife test was used to measure the significant contributions of the BIOCLIM variables on the occurrence prediction for each model. Finally, potential distribution ranges for the present-day and LGM were projected in ArcMap 9.3 (ESRI, Redlands, CA, USA).

## 3. Results

### 3.1. Chloroplast Variation and Haplotype Geographical Distribution

**Figure 1:**
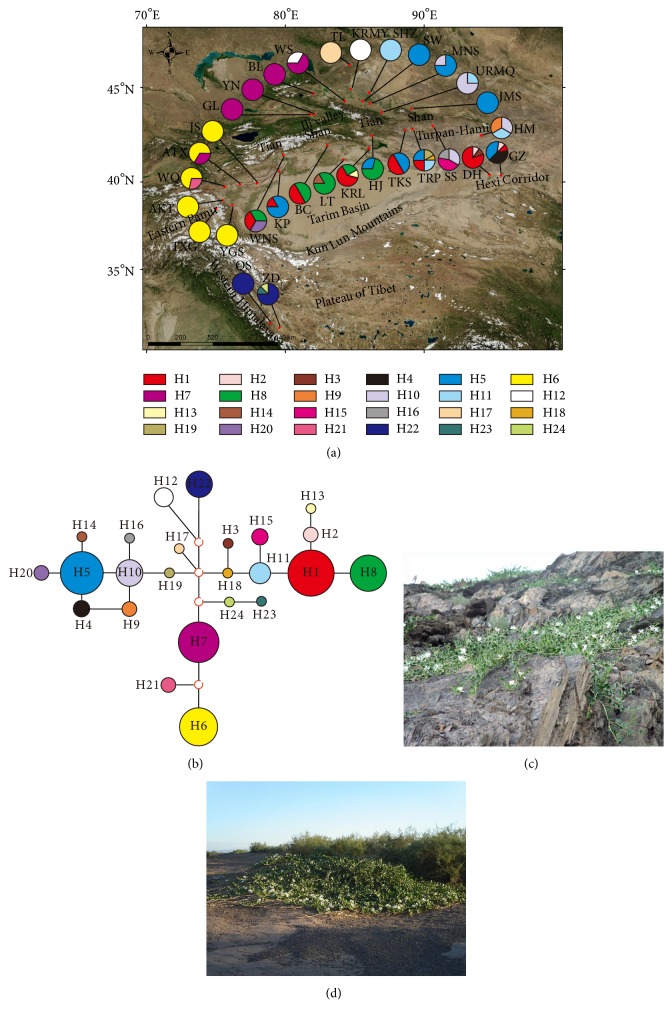
Geographical distribution and phylogenetic network of haplotypes in sampled locations of* Capparis spinosa*. (a) Distribution of the cpDNA haplotypes. The abbreviated letters of population localities correspond to those shown in Table 1. Pie charts show the different haplotypes and their proportions in each population; different colors are consistent with matching haplotypes in the figure legend. (b) Median-joining network reflecting the cpDNA haplotype relationships. Small, open red circles represent potentially intermediate genotypes with other mutational steps between real haplotypes; circle sizes are proportional to haplotype frequencies. (c, d) Two different kinds of habitats of the species: (c) eroding mountain slopes and (d) gravel deserts.

The aligned sequence length of the three cpDNA regions was 2254 bp (923 bp for* trn*L-*trn*F, 728 bp for* rps*12-*rpl*20, and 603 bp for* ndh*F (329F, 927R)). Based on the total sequences, thirty-six polymorphic sites (thirty nucleotide substitutions and six indels) were obtained (Table S2). A total of 24 haplotypes (H1–H24) were identified from 267 samples among 31 populations across the entire geographic range of* C. spinosa* (Figure 1(a), Table 1).

**Figure 2:**
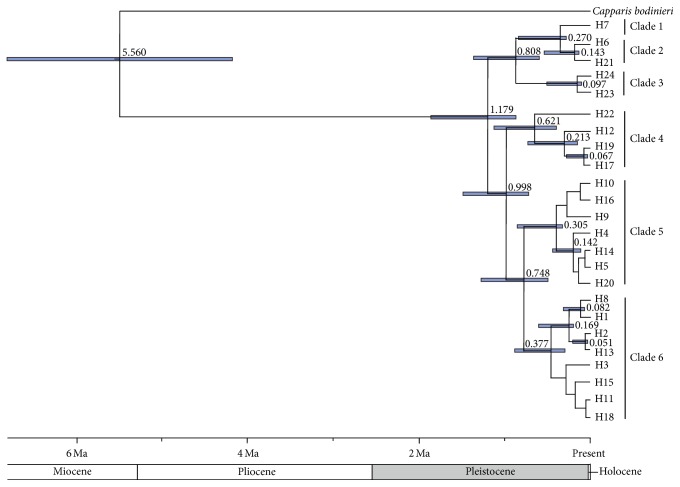
Phylogenetic chronogram of 24 haplotypes in* C. spinosa* generated from BEAST analysis. Divergence time (million years ago) of nodes are shown; blue horizontal bars indicating the 95% ranges of highest posterior density.

According to the results of SAMOVA, the 31 studied populations were divided into five defined phylogeographical groups: (I) the Western Himalayas Group, (II) the Eastern Pamir Group, (III) the Ili Valley Group, (IV) the north side of the Tianshan Mountains Group, and (V) the south side of the Tianshan Mountains Group (Table 1). The main genealogical relationships of the network connection diagram (Figure 1(b)) as well as the BEAST tree (Figure 2) suggested the similar divergence trend.

Distribution of the haplotypes showed high geographic structure. Haplotypes H22, H6, H7, H1, H8, H5, and H10 had the most frequent and widespread distributions: H22 only was distributed in the populations of Group I; H6 occurred in all populations of Group II; H7 only occurred in the populations of Group III; H1 and H8 were dominantly distributed in the populations of Group V, while H5 and H10 were mainly present in the populations of Group IV (Figure 1(a)).

### 3.2. Genetic Diversity and Population Genetic Structure

Genetic diversity analysis revealed that total genetic diversity (*H*_*T*_ = 0.916, s.e. = 0.0172) was much higher than average gene diversity (*H*_*S*_ = 0.316, s.e. = 0.0533), which indicated that there was considerable population differentiation across the distributional range. The *U* Test showed that total *N*_ST_ (0.843, s.e. = 0.033) was significantly higher than *G*_ST_ (0.655, s.e. = 0.057) (*U* = 6.91, *P* < 0.01), indicating cpDNA variation with signal phylogeographic structure for the species.

AMOVA revealed that a larger proportion of the variation was interpopulational than intrapopulational (85.20% versus 14.80%). Differences among geographical groups were significant, and AMOVA results showed that 71.83% (*F*_CT_ = 0.71831, *P* < 0.001) of total variation existed between the five groups (Table 2). For the two genetic diversity indexes, haplotype diversities (*H*_*d*_) within the 31 populations ranged from 0 to 0.8485, and nucleotide diversities (*π*) ranged from 0 to 0.0021. Considering the whole species, high degrees of haplotype diversity (*H*_*d*_ = 0.8925) and nucleotide diversity (*π* = 0.0037) were found. At the level of population, six populations (TRP, HM, WNS, GZ, SS, and KRL) in Group V showed considerably high degrees of genetic diversity (Table 1).

### 3.3. Molecular Divergence Time Estimates and Demographic History Analyses

The phylogenetic tree yielded by BEAST analysis was strongly coupled with geography, demonstrating that the coalescent sequences were suitable to be employed in phylogenetic and phylogeographic analysis. The results indicate that the identified cpDNA haplotypes are clustered into six main clades. Divergence times estimated from BEAST analysis were Pleistocene, and the main divergence between the geographical groups began at 1.179 Ma, in early Pleistocene (Figure 2).

**Figure 3:**
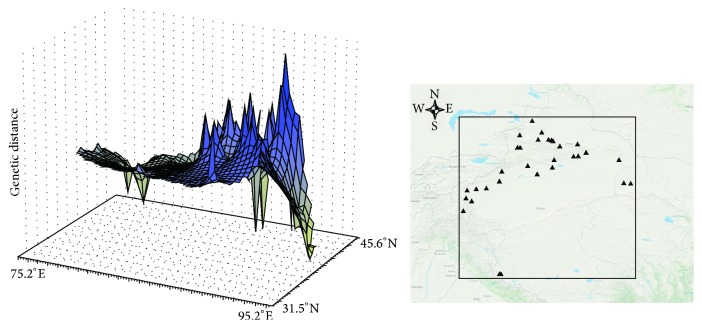
Spatial genetic landscape shapes constructed by interpolation analysis for* C. spinosa*. The abscissae and ordinates correspond to geographical coordinates covering the entire distributional populations, and the vertical axes represent genetic distances.

**Figure 4:**
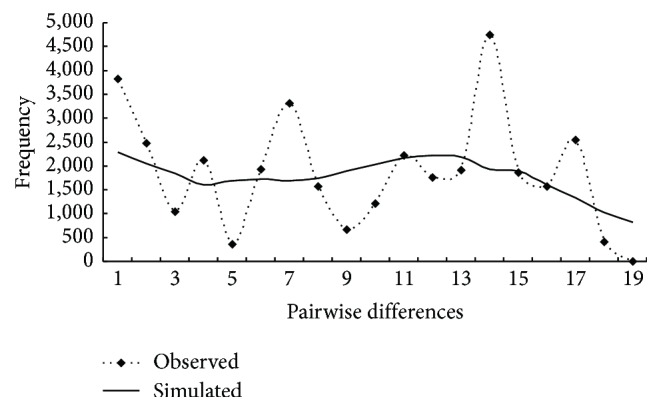
Pairwise mismatch distributions of cpDNA sequences of* C. spinosa.*

The three-dimensional surface plot produced by genetic landscape shape analyses showed that spatial genetic distances gradually increased from the western populations to eastern populations (Figure 3). For mismatch analysis, the observed mismatch curve was not unimodal but strongly discordant with a model of sudden range expansion (Figure 4), indicating the stable populations. Additionally, no supporting evidence was provided by Tajima's *D* and Fu's *F*_*s*_ values for recent range expansion events.

### 3.4. Potential Distribution Modeling

**Figure 5:**
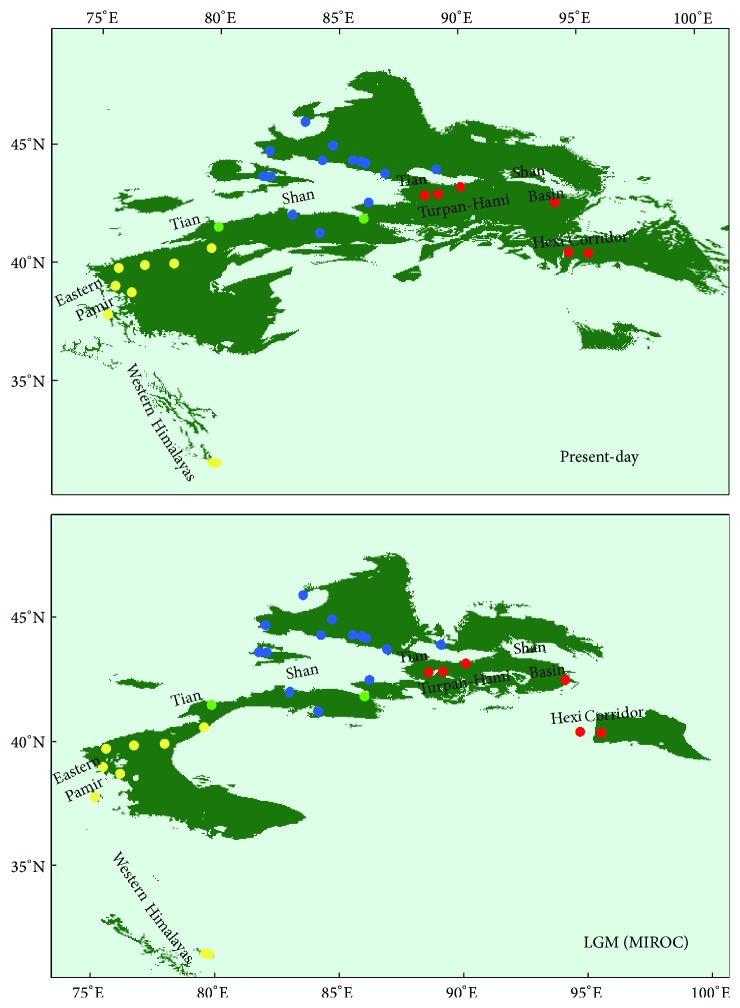
Species distribution modeling of* C. spinosa* during present-day and LGM periods in arid Eastern Central Asia. Potential distribution in the LGM epoch is based on scenarios of the MIROC model. Yellow localities: populations sensitive to glaciation in the high mountains. Blue localities: populations at middle elevations. Green localities: gorges served as refugia in the mid-Tianshan. Red localities: populations of low-altitude desert region.

Under the selected model, the test AUC value for the ENM of* C. spinosa* in the present-day was 0.995; and, under the MIROC climate scenario, the value was 0.997 in the LGM projection. SDM did not yield suitable or credible distribution in LGM based on the CCSM model. The Jackknife analysis of regularized training gain indicated that there were five environmental variables contributing more highly to the distribution model in the present-day: precipitation of the wettest month (22.64%), mean temperature of the coldest quarter (22.97%), precipitation of the coldest quarter (15.48%), mean temperature of the driest quarter (8.25%), and precipitation seasonality (6.37%). The five most contributive bioclimatic variables of the MIROC model in the LGM were annual mean temperature (24.32%), precipitation of the coldest quarter (20.18%), mean temperature of the coldest quarter (13.87%), mean temperature of the driest quarter (12.18%), and precipitation of the wettest month (11.89%). The range differences under the distinct climatic conditions of the two models revealed a diminished amount of suitable habitat for the species during the LGM period. In comparison with the present potential distribution, the MIROC scenario indicated that the high-altitude ranges of the species in the Western Himalayas, eastern Pamir, and western Tianshan Mountains were diminished (Figure 5), according to the LGM modeling distribution. However, some localities in the middle Tianshan, such as WNS and KRL, were not affected by this in the LGM model (Figure 5), and thus midelevation gorges environments might have provided suitable survival habitats for the species in the glacial epoch.

## 4. Discussion

### 4.1. Genetic Variation

At the species level, a high total gene diversity (*H*_*T*_ = 0.916) was detected. Compared with other species where three intergenic spacers have been sequenced, gene diversity in* C. spinosa* was higher, for instance,* Juniperus sabina* (*H*_*T*_ = 0.577) [70] and* Gymnocarpos przewalskii* (*H*_*T*_ = 0.903) [29]. The results of genetic diversity analysis also give indication of a high level of total haplotype diversity (*H*_*d*_ = 0.8925) (Table 1). High values of the diversity indices might have been promoted by the accumulation of variation in* C. spinosa,* which, to a great extent, may have been associated with differences in geological and topographical conditions, complex habitat patterns, and gradually intensive aridification occurring in Eastern Central Asia during the Quaternary period.

Dramatic genetic variation among the total populations was shown, accounting for a far greater proportion than that within populations (Table 2). The genetic differences among populations are believed to be closely related to the degree of gene exchange [71], which depends on seed transfer for cpDNA in most angiosperms [72]. The seed dispersal modes of* C. spinosa* mainly include anemochory, myrmecochory, and endozoochory by rodent vector [73]. In the current survey regions, the mountains and sprawling desert zones isolated habitat into disjunctive geographical units. In this situation, gene flow between populations was potentially restricted in short distance. Especially in small sized populations, individuals might be susceptible to inbreeding. Therefore, gene flow was limited attributing to the incapacity of seed dispersal across impassable physical barriers by anemochory or zoochory, resulting in population differentiation.

### 4.2. Influence of Pleistocene Geological and Climatic Changes on Phylogeographical Structure

The timescales of intraspecific divergences are usually related to historical geologic and climatic events [3, 12]. The estimated genetic divergence time between the five geographical groups started at approximately 1.179 million years ago based on molecular dating results of the BEAST analysis (Figure 2), which falls into early Pleistocene, the phase of intense uplift of Himalayas [74]. From early to middle Pleistocene, most large-scale piedmont glaciers developed on the northern slope of the Himalayas [75], which may have been the impetus causing haplotypes H23, H24, and H22 belonging to the Western Himalayan populations to branch off the phylogenetic tree (0.808–0.621 Ma) (Figure 2). At the intersection of the Himalaya, Kunlun, and Tianshan Mountains, the Pamir uplift was structured from at least the end of the Paleogene. In the Quaternary stage, owing to at least three large-scale glaciations, piedmont moraines intermittently formed in this region [76]. In connection with these fluctuations, the Western Himalayan and Eastern Pamirian populations from high-altitude localities experienced large-scale differentiation, accompanied with adaptation to colder climates, resulting in different degrees of genetic diversity and conspicuous population differences. We found that haplotypes H22 and H6 were, respectively, distributed over every sampled location in Western Himalayan and Eastern Pamir region, while the rare haplotypes H23, H24, and H21 were randomly distributed in single populations. Although BEAST analysis and network diagram did not clearly show that haplotype H22 clustered into the same clade with H23 and H24, it occupies a key transitional position (Figures 1(b) and 2). Genetically H22 contained a haplotype approximating those in the Tianshan region, while geographically it is distributed in the Western Himalayan region, indicating a possible common Tethyan origin and then allopatric divergence and local evolution.

In comparison with the formation of the Himalayas and Pamirs, neotectonic movement of Tianshan Mountains was somewhat lagging, occurring in Pliocene and intensively uplifting in early Pleistocene [7, 8]. Since Quaternary diastrophism and climatic changes, together with more than three glacial-interglacial cycles [77], not only changed the topography but also resulted in temperature and precipitation differences between western and eastern ranges, north and south slopes, and high and low altitudes [74], rapid uplift of the Tianshan range as a barrier would, of necessity, have changed the local atmospheric circulation and screened the sources of moist airflow, intensifying the arid climate in adjacent regions. Later, since the mid-Pleistocene, enhanced drought and cold occurred in the Tianshan and the surroundings at 0.6–0.2 Ma [78], as evidenced by loess sediments on northern slope of the Tianshan Mountains [74]. During this period, a weakened southwest monsoon and strengthened plateau winter monsoon resulted in increasing aridification and desert expansion [79]. Compared with the geographic patterns under present-day climatic conditions, distribution ranges of* C. spinosa* during the LGM period were contracted (Figure 5). Although distribution areas of the species changed, the main distribution in the low-altitude eastern desert region was stable during glacial periods and only marginally influenced. The great spatial and genetic distances among locations in the eastern Tianshan and Hexi Corridor can be perceived in the 3D surface plots (Figure 3). This characteristic of the spatial genetic landscape analysis also demonstrates that the species has had relatively stable habitats in the low-altitude desert region during an extensive evolutionary history. At the stage of the mid-Pleistocene period, the continuously expanding Taklimakan [80] and intensive aridification accelerated the worsening drought and heat for the lower elevations of the eastern Tianshan, Turpan-Hami Basin, and Hexi Corridor populations, which must have influenced recent divergence among these areas. In general, genetic divergence among geographic groups in Pleistocene appears to have been driven mainly by intense uplift of the Himalayas and Tianshan Mountains. The cold-dry to warm-humid climatic cycles during the late Quaternary are inferred to have promoted genetic divergence within groups.

### 4.3. Potential Glacial Refugia and Demographic Dispersal in Past Scenarios

According to previous glaciological research, maximum glaciation was likely to have occurred in the early phase of the mid-Pleistocene (0.8–0.6 Ma) in Eastern Central Asia [74, 77]. During this period and the next two glacial stages, owing to large-scale glacial advances, glaciers in Tianshan at one time extended as far down as the piedmont belts [74]. In comparison with the present distribution range, the LGM localities of* C. spinosa* were reduced in the high-altitude ranges of the Western Himalayas, Pamirs, and western Tianshan Mountains (Figure 5), as a result of frigid climate in the wake of glacial advances. The increasingly severe climate in the glacial epoch would have damaged the ecological habitats of some xeric plant species [81]; however, it seems likely that* C. spinosa* could have survived in some stable gorges of Tianshan Mountains. These suitable refugia were less influenced by climatic oscillations and therefore could maintain richer degrees of genetic diversity among populations [25, 82]. Refugial zones are generally recognizable because of high levels of genetic diversity and unique genotypes in species populations [12, 22] and are usually located in the middle altitude of mountains, which could supply warmer habitats for species to resist cold glacial climates [83]. A number of glacial refugia have been revealed worldwide on the basis of molecular approaches [84–86]. However, refugia localities in the Tianshan and adjacent arid regions have remained rather cryptic, as there seems to be a lack of applicable studies [23]. As a representative xerophyte,* C. spinosa* selected relatively warm gorges (WNS and KRL) in the middle Tianshan Mountains as potential refugia during glacial epochs, in accordance with the high levels of genetic diversity within these populations.

Unlike the traditional northward expansions during warm interglacial periods [18, 87], in the current study, the dispersal of* C. spinosa* was profoundly affected not only by temperature variation but also by aridification in Eastern Central Asia; thus xeric species may well prefer relatively arid areas during the warm and moist interglacial stages. Considering the network diagram, genotypes H1 and H8 were found with high rates of incidence on the south side of Tianshan group, indicating that these two were predominant haplotypes in Group V (Figure 1). These characteristics imply that the species should have undergone large-scale interglacial expansions from glacial refugia in this region. However, multimodal mismatch distribution shape (Figure 4) and positive Fu's *F*_*s*_ value failed to provide any significant evidence for recent range expansion. According to Printzen et al. (2003), extensive intraspecific differentiation is probably connected with range fragmentation or long-distance dispersal [88]. Evidenced from our field observations,* C. spinosa* is a xeromorphic species that prefers to distribute itself in eroding rocky mountains and arid piedmont proluvial or alluvial gravelly fans (Figures 1(c) and 1(d)). In the end of early Pleistocene and the early mid-Pleistocene, intensified aridification and the formation of Taklimakan desert resulted in dry and rainless conditions in piedmont zones of Tianshan [74, 89]. However, in contrast with the cold climate in glacial phase, in the interglacial and postglacial period the climate became warm and humid. At this time, glacier-melt water increased the volume of river runoff on the edge of the Tarim Basin [74]. Thus, the movement of the wind and river runoff provided appropriate conditions for survival and seed dispersal of* C. spinosa*. These could harbor relatively stable habitats, preserving the species' existing genetic variation and genetic differences among populations. There is considerable evidence, such as increasing *δ* ^18^ *O* values, palynological components, multiple alluvial sequences, and lake sediments, that reveals the alternation of glacials and interglacials occurring in the Tianshan Mountains during the Pleistocene [74, 90, 91]. This circularly fluctuant climate propelled demographic dispersal in the interglacial intervals. Interestingly, unlike most other species with decreased levels of genetic diversity related to desert expansion during interglacial and postglacial colonization [29, 30], as xeric Tethyan relic,* C. spinosa* showed successively increased levels of genetic diversity and population sizes, especially when it contacted the more droughty and hot gravelly deserts in Turpan-Hami Basin and Hexi Corridor. One reasonable explanation for this phenomenon is the probability that the suitable warm temperate continental climate in eastern gravel deserts is similar to the ancient hot and dry subtropical summer climate of the Tethyan Flora, preserving the characteristics of the original climate and environment. The other corollary is that the desert region could play the role of a shelter influenced only marginally by glaciation.

## 5. Conclusions

The current study focuses on how complicated Pleistocene geological and cyclical climatological events influenced the phylogeographic structure and genetic divergence of* C. spinosa* in arid Eastern Central Asia. Strongly spatial phylogeographic patterns were documented in the species. Intense uplift of the Himalaya and Tianshan Mountains around the early Pleistocene, coupled with a cold-dry climate and consequent aridification during the stage of Quaternary glaciation in this survey region, played important roles both in triggering and in shaping the current phylogeographic structure of the species group. There were at least three glacial-interglacial cycles in the Himalayas, as well as the Pamir and Tianshan Mountains during the Pleistocene. During glacial epochs, potential refugia were inferred for the gorges of the middle Tianshan Mountains, while, in interglacials and the postglacial period, the species experienced dispersal. The phylogeography of* C. spinosa* in the present research provides the basis for future studies on how xerophilous plant species across both arid high-altitude rocky mountains (Figure 1(c)) and low-altitude gravel deserts (Figure 1(d)) responded in a stepwise fashion to Quaternary geologic and climatic events.

## Supplementary Material

Supplementary Table S1: Universal primers used for screening sequence variation at population level of *Capparis spinosa*.
Supplementary Table S2: Variable sites of three chloroplast DNA sequences (*trn*L-*trn*F, *rps*12-*rpl*20, and *ndh*F) in twenty-four haplotypes of *Capparis spinosa*.

## Figures and Tables

**Table 1:** Details of geographical locations, sample sizes, cpDNA haplotypes, and diversity indices for 31 populations of *C. spinosa*.

Code	Population locality	Latitude/longitude	Altitude (m)	No.	Haplotype	*H*_*d*_ (±SD)	*π* (±SD)
Overall				267		0.8925 ± 0.0075	0.0037 ± 0.0019

(I) The Western Himalayas group							
(1) ZD	Zanda, Tibet	31°30′N/79°38′E	3,598	9	H22, H23, H24	0.4167 ± 0.1907	0.0021 ± 0.0013
(2) QS	Qusum, Tibet	31°29′N/79°48′E	3,645	5	H22	0	0

(II) The Eastern Pamir group							
(3) YGS	Yengisar, Xinjiang	38°43′N/76°14′E	2,010	9	H6	0	0
(4) TXG	Taxkorgan, Xinjiang	37°47′N/75°13′E	3,694	5	H6	0	0
(5) AKT	Akto, Xinjiang	38°59′N/75°32′E	2,441	9	H6	0	0
(6) WQ	Wuqia, Xinjiang	39°44′N/75°40′E	1,945	10	H6, H21	0.4667 ± 0.1318	0.0006 ± 0.0005
(7) ATX	Artux, Xinjiang	39°52′N/76°47′E	1,303	10	H6, H7	0.4667 ± 0.1318	0.0006 ± 0.0005
(8) JS	Jiashi, Xinjiang	39°56′N/78°01′E	1,162	9	H6	0	0

(III) The Ili Valley group							
(9) GL	Gongliu, Xinjiang	43°37′N/81°49′E	710	9	H7	0	0
(10) YN	Yining, Xinjiang	43°37′N/82°08′E	750	9	H7	0	0
(11) BL	Bole, Xinjiang	44°42′N/82°05′E	470	9	H7	0	0
(12) WS	Wusu, Xinjiang	44°19′N/84°19′E	685	9	H7, H12	0.5000 ± 0.1283	0.0013 ± 0.0009

(IV) The north side of the Tianshan Mountains group							
(13) KRMY	Karamay, Xinjiang	44°56′N/84°46′E	296	5	H12	0	0
(14) TL	Toli, Xinjiang	45°55′N/83°36′E	684	5	H17	0	0
(15) SHZ	Shihezi, Xinjiang	44°16′N/85°57′E	356	5	H11	0	0
(16) SW	Shawan, Xinjiang	44°19′N/85°35′E	584	9	H5	0	0
(17) MNS	Manas, Xinjiang	44°11′N/86°08′E	642	8	H5, H10	0.4286 ± 0.1687	0.0002 ± 0.0002
(18) URMQ	Urumqi, Xinjiang	43°44′N/86°57′E	1,060	8	H10, H11	0.4286 ± 0.1687	0.0010 ± 0.0007
(19) JMS	Jimsar, Xinjiang	43°55′N/89°07′E	945	8	H5	0	0

(V) The south side of the Tianshan Mountains group							
(20) KP	Kalpin, Xinjiang	40°35′N/79°36′E	1,131	10	H1, H5	0.3556 ± 0.1591	0.0008 ± 0.0006
(21) WNS	Wensu, Xinjiang	41°29′N/79°54′E	1,296	9	H1, H8, H20	0.7500 ± 0.0786	0.0016 ± 0.0010
(22) BC	Baicheng, Xinjiang	41°59′N/83°03′E	1,369	8	H1, H8	0.5714 ± 0.0945	0.0003 ± 0.0003
(23) LT	Luntai, Xinjiang	41°14′N/84°12′E	921	10	H8, H14	0.3556 ± 0.1591	0.0011 ± 0.0007
(24) KRL	Korla, Xinjiang	41°50′N/86°03′E	1,072	8	H1, H8, H13	0.6071 ± 0.1640	0.0004 ± 0.0004
(25) HJ	Hejing, Xinjiang	42°31′N/86°16′E	1,403	10	H5, H8	0.4667 ± 0.1318	0.0012 ± 0.0008
(26) TKS	Toksun, Xinjiang	42°49′N/88°38′E	95	12	H1, H5	0.5455 ± 0.0615	0.0012 ± 0.0008
(27) TRP	Turpan, Xinjiang	42°52′N/89°11′E	−84	12	H1, H5, H11, H18, H19	0.8485 ± 0.0586	0.0017 ± 0.0010
(28) SS	Shanshan, Xinjiang	43°11′N/90°09′E	1,043	9	H10, H15, H16	0.7222 ± 0.0967	0.0015 ± 0.0009
(29) HM	Hami, Xinjiang	42°31′N/94°09′E	773	9	H9, H10, H11	0.7500 ± 0.0786	0.0013 ± 0.0009
(30) DH	Dunhuang, Gansu	40°25′N/94°44′E	1,051	10	H1, H2, H3	0.3778 ± 0.1813	0.0005 ± 0.0004
(31) GZ	Guazhou, Gansu	40°22′N/95°34′E	1,133	10	H1, H2, H4, H5	0.7333 ± 0.1005	0.0011 ± 0.0007

No.: number of sample individuals; *H*_*d*_: haplotype diversity; *π*: nucleotide diversity.

**Table 2:** Results of AMOVA analysis of molecular variance for populations and geographical groups of *C. spinosa*.

Source of variation	d.f.	SS	VC	PV (%)	Fixation index
Among groups	4	738.961	3.723	71.83	*F*_CT_: 0.71831^*∗∗*^
Among populations within groups	26	175.341	0.693	13.37	*F*_SC_: 0.47458^*∗∗*^
Within populations	236	181.039	0.767	14.80	*F*_ST_: 0.85199^*∗∗*^
Total	266	1095.341	5.183		

d.f.: degrees of freedom; SS: sum of squares; VC: variance components; PV: percentage of variation; *F*_SC_: correlation of chlorotypes within populations relative to groups; *F*_ST_: correlation within populations relative to the total; *F*_CT_: correlation within groups relative to the total; ^*∗∗*^ *P* < 0.001; 1000 permutations.
