# Supplementary material for: Phylogeographic Structure of a Tethyan Relict Capparis spinosa (Capparaceae) Traces Pleistocene Geologic and Climatic Changes in the Western Himalayas, Tianshan Mountains, and Adjacent Desert Regions
Source: Biomed Res Int. 2016 May 24;2016:5792708. doi: 10.1155/2016/5792708 (PMC4903145; doi:10.1155/2016/5792708)
Supplement: Supplementary file 1 — Supplementary Table S1: Universal primers used for screening sequence variation at population level of Capparis spinosa. Supplementary Table S2: Variable sites of three chloroplast DNA sequences (trnL-trnF, rps12-rpl20, and ndhF) in twenty-four haplotypes of Capparis spinosa. [file 5792708.f1.doc]

# TABLE S1: Universal primers used for screening sequence variation at population level of *Capparis spinosa*.

| **Region** | **Primer sequence** | **Reference** |
| --- | --- | --- |
| ITS | ITS1: TCCGTAGGTGAACCTGCGG; ITS2: GCTGCGTTCTTCATCGATGC | White *et al*. (1990) |
| *psb*A–*trn*H | F: GTTATGCATGAACGTAATGCTC; R: CGCGCATGGTGGATTCACAATCC | Sang *et al*. (1997) |
| *trn*S–*trn*G | F: GCCGCTTTAGTCCACTCAGC; R: GAACGAATCACACTTTTACCAC | Hamilton (1999) |
| *psb*K–*psb*I | F: TTAGCCTTTGTTTGGCAAG; R: AGAGTTTGAGAGTAAGCAT | Lahaye *et al.* (2008) |
| *trn*L–*trn*F | F: CGAAATCGGTAGACGCTACG; R: ATTTGAACTGGTGACACGAG | Taberlet *et al*. (1991) |
| *psb*B-*psb*F | F: GTTTACTTTTGGGCATGCTTCG ; R: CGCAGTTCGTCTTGGACCAG | Hamilton (1999) |
| *rpo*B–*trn*C | F: CACCCRGATTYGAACTGGGG; R: CKACAAAAYCCYTCRAATTG | Shaw *et al*. (2005) |
| *trn*Q–*rps*16 | F: GCGTGGCCAAGYGGTAAGGC; R: GTTGCTTTYTACCACATCGTTT | Shaw *et al*. (2007) |
| *rps*16 | F:AAACGATGTGGTARAAAGCAAC; R: AACATCWATTGCAASGATTCGATA | Shaw *et al*. (2005) |
| *rps*16–*trn*K | F: AAAGTGGGTTTTTATGATCC; R: TTAAAAGCCGAGTACTCTACC | Shaw *et al*. (2007) |
| *ycf*6–*psb*M | F: TGGATATAGTAAGTCTYGCTTGGGC; R: AGTGCATGGAGTCTYGCTAGG | Shaw *et al*. (2007) |
| *Trn*D–*trn*T | F: ACCAATTGAACTACAATCCC; R: CTACCACTGAGTTAAAAGGG | Shaw *et al*. (2005) |
| *trn*T–*trn*L | F: CAAATGCGATGCTCTAACCT; R:TCTACCGATTTCGCCATATC | Shaw *et al*. (2005) |
| *rps*12–*rpl*20 | F: ATTAGAAANRCAAGACAGCCAAT; R: CGYYAYCGAGCTATATATCC | Shaw *et al*. (2005) |
| *rpl*32–*trn*L | F: CAGTTCCAAAAAAACGTACTTC; R: CTGCTTCCTAAGAGCAGCGT | Shaw *et al*. (2007) |
| *ndh*F–*rpl*32 | F: GAAAGGTATKATCCAYGMATATT; R: CCAATATCCCTTYYTTTTCCAA | Shaw *et al*. (2007) |
| *ndh*F (3’ end) | 972F: GTCTCAACTCGGTTATATGATG ; 2110R: CCACCTATATATTTTGTTACTTCTCC | Hall *et al*. (2002) |
| *ndh*F(329F, 927R)* | F: CGTTCTATTCCATCTCTCTATG; R: AGCATCTATGTAACCACGA |  |

F, forward; R, reverse; *, *ndh*F (329F, 927R) primers were designed by Primer Premier 5.0 software (<http://www.PremierBiosoft.com/primerdesign/primerdesign.html>) and evaluated by Oligo 6 program (http://www.oligo.net/) based on *ndh*F sequence of *Capparis spinosa* from GenBank (EU373694).

# TABLE S2: Variable sites of three chloroplast DNA sequences (trnL-trnF, rps12-rpl20, and ndhF) in twenty-four haplotypes of C. spinosa.

| Haplotype | Sequence position | | | | | | | | | | | | | | | | | | | | | | | | | | | | | | | | | |
| --- | --- | --- | --- | --- | --- | --- | --- | --- | --- | --- | --- | --- | --- | --- | --- | --- | --- | --- | --- | --- | --- | --- | --- | --- | --- | --- | --- | --- | --- | --- | --- | --- | --- | --- |
|  |  |  |  | 1 | 1 | 1 | 1 | 1 | 1 | 1 | 1 | 1 | 1 | 1 | 1 | 1 | 1 | 1 | 1 | 1 | 1 | 1 | 1 | 2 | 2 | 2 | 2 | 2 | 2 | 2 | 2 | 2 | 2 | 2 |
|  | 2 | 3 | 9 | 1 | 3 | 7 | 7 | 7 | 8 | 8 | 8 | 8 | 8 | 8 | 9 | 9 | 9 | 9 | 9 | 9 | 9 | 9 | 9 | 0 | 0 | 0 | 0 | 0 | 1 | 1 | 1 | 1 | 1 | 1 |
|  | 8 | 1 | 9 | 4 | 7 | 1 | 5 | 6 | 0 | 0 | 1 | 1 | 3 | 5 | 0 | 1 | 4 | 4 | 5 | 6 | 8 | 8 | 9 | 3 | 4 | 8 | 8 | 9 | 0 | 4 | 5 | 8 | 9 | 9 |
|  | 5 | 0 | 8 | 4 | 2 | 0 | 3 | 4 | 2 | 9 | 1 | 7 | 2 | 2 | 7 | 2 | 0 | 9 | 0 | 9 | 4 | 8 | 5 | 0 | 7 | 5 | 9 | 5 | 9 | 0 | 6 | 8 | 7 | 9 |
| H1 | T | – | C | A | A | T | C | G | A | T | C | G | C | G | – | C | C | C | A | C | A | T | C | C | T | C | T | G | T | T | A | G | G | G |
| H2 | T | – | C | A | A | T | C | G | A | T | C | G | C | G | – | C | C | C | A | C | A | T | C | A | T | C | T | G | T | T | A | G | G | G |
| H3 | A | ★ | C | A | A | T | C | G | A | T | C | G | C | G | – | C | C | C | A | C | A | T | C | A | T | C | T | G | T | T | A | G | G | G |
| H4 | T | – | C | A | A | T | C | G | A | T | T | G | A | G | T | C | C | C | A | C | G | T | C | C | T | C | C | G | T | T | A | T | G | G |
| H5 | T | – | C | A | A | T | C | G | A | T | T | G | A | G | T | C | C | C | A | C | G | T | C | C | T | C | T | G | T | T | A | T | G | G |
| H6 | A | ★ | C | A | G | T | C | G | G | T | C | A | C | G | – | C | T | C | A | T | G | T | C | C | T | C | T | G | C | G | G | G | G | G |
| H7 | A | ★ | C | A | G | T | C | G | A | T | C | A | C | G | – | C | T | C | A | C | G | T | C | C | T | C | T | G | T | G | G | G | G | G |
| H8 | T | – | C | A | A | T | C | G | A | T | C | G | C | G | – | G | C | C | A | C | A | T | C | C | T | C | T | G | T | T | A | G | G | G |
| H9 | A | – | C | A | A | T | C | G | A | T | T | G | A | G | T | C | C | C | A | C | G | T | C | C | T | C | C | G | T | T | A | T | G | G |
| H10 | A | – | C | A | A | T | C | G | A | T | T | G | A | G | T | C | C | C | A | C | G | T | C | C | T | C | T | G | T | T | A | T | G | G |
| H11 | A | – | C | A | A | T | C | G | A | T | C | G | C | G | – | C | C | C | A | C | A | T | C | C | T | C | T | G | T | T | A | G | G | G |
| H12 | A | ★ | C | A | G | T | C | G | A | C | C | G | C | G | – | C | C | C | A | C | G | T | C | C | T | G | T | G | T | T | A | G | G | G |
| H13 | T | – | C | A | A | T | C | G | A | T | C | G | C | G | – | C | C | T | A | C | A | T | C | A | T | C | T | G | T | T | A | G | G | G |
| H14 | T | – | C | A | A | T | C | G | A | T | T | G | A | G | T | C | C | C | G | C | G | T | C | C | T | C | T | G | T | T | A | T | G | G |
| H15 | A | – | C | C | A | T | C | G | A | T | C | G | C | G | – | C | C | C | A | C | A | T | C | C | T | C | T | G | T | T | A | G | G | G |
| H16 | A | – | C | C | A | T | C | G | A | T | T | G | A | G | T | C | C | C | A | C | G | T | C | C | T | C | T | G | T | T | A | T | G | G |
| H17 | A | – | C | A | A | T | C | G | A | C | C | G | C | G | – | C | C | C | A | C | G | T | C | C | T | G | T | G | T | T | A | G | G | G |
| H18 | A | ★ | C | A | A | T | C | G | A | T | C | G | C | G | – | C | C | C | A | C | A | T | C | C | T | C | T | G | T | T | A | G | G | G |
| H19 | T | – | C | A | A | T | C | G | A | C | C | G | C | G | – | C | C | C | A | C | G | T | C | C | T | G | T | G | T | T | A | G | G | G |
| H20 | T | – | C | A | A | T | C | G | A | T | T | G | A | G | T | C | C | C | A | C | G | T | C | C | C | C | T | G | T | T | A | T | G | G |
| H21 | A | ★ | C | A | G | T | C | G | G | T | C | A | C | G | – | C | T | C | A | C | G | A | C | C | T | C | T | G | T | G | G | G | G | G |
| H22 | A | ★ | G | A | G | A | – | C | A | C | C | G | C | G | – | C | C | C | A | C | G | T | C | C | T | C | T | G | T | T | A | G | A | G |
| H23 | T | – | G | A | G | T | C | G | A | T | C | A | C | A | – | C | C | C | A | C | G | T | – | C | T | C | T | A | T | T | A | G | G | C |
| H24 | A | ★ | G | A | G | T | C | G | A | T | C | A | C | A | – | C | C | C | A | C | G | T | – | C | T | C | T | A | T | T | A | G | G | C |

★，ATA.
